# Pubic Symphysis Rupture During the Second Stage of Labor: A Case Report

**DOI:** 10.7759/cureus.103780

**Published:** 2026-02-17

**Authors:** Alexander M Harrison, Olivia F Zucaro, Jacob Speybroeck, Ari Levine, Johnathan B Glabb, Angela C Ranzini

**Affiliations:** 1 Obstetrics and Gynecology, MetroHealth Medical Center, Cleveland, USA; 2 Obstetrics and Gynecology, University of Kentucky College of Medicine, Lexington, USA; 3 Orthopedic Surgery, Case Western Reserve University School of Medicine, Cleveland, USA; 4 Orthopedic Surgery, MetroHealth Medical Center, Cleveland, USA; 5 Radiology, MetroHealth Medical Center, Cleveland, USA; 6 Obstetrics and Gynecology, MetroHealth Medical Center/Case Western Reserve University School of Medicine, Cleveland, USA

**Keywords:** orthopedics, peripartum complication, pregnancy, pubic symphysis, pubic symphysis rupture

## Abstract

A multiparous woman underwent induction of labor and progressed to the second stage. During the second stage, an audible "pop" was heard. She developed significant pain and edema. Through a multidisciplinary approach, the patient was diagnosed with a pubic symphysis rupture. Imaging demonstrated a separation of the pubic symphysis of 8.5 cm. By the end of the multidisciplinary care, this distance had been reduced to 3 cm, and the patient was back to baseline function following treatment. This case demonstrates and encourages the use of a multidisciplinary team including radiology, trauma, orthopedics, and obstetrics. A high level of suspicion and effective interdisciplinary communication can result in optimal outcomes for this rare complication, as is seen in this patient.

## Introduction

Physiologic changes during pregnancy, including increased levels of relaxin and progesterone, result in softening of the pelvic ligaments and increased mobility of the pelvic girdle to facilitate vaginal delivery. While mild widening of the pubic symphysis is expected in pregnancy, separation greater than 1 cm is generally considered pathologic and may be associated with significant pain, instability, and functional limitation [1]. The pubic symphysis is a fibrocartilaginous joint located at the anterior midline of the pelvis, connecting the left and right pubic bones and contributing to overall pelvic ring stability. During pregnancy, the hormonal influences, such as increased levels of relaxin and progesterone, increase ligamentous laxity to facilitate delivery; however, excessive separation may compromise stability and function. Pubic symphysis diastasis and rupture are rare complications of vaginal delivery, with reported incidence ranging widely from one in 300 to one in 30,000 deliveries, reflecting differences in diagnostic criteria and reporting practices [2-4]. Although separation greater than 1 cm is considered pathologic, larger thresholds (often cited as greater than 4 cm) are typically discussed in the context of operative management rather than diagnosis [4].

Risk factors associated with peripartum pubic symphysis rupture include multiparity, fetal macrosomia, precipitous or prolonged second stage of labor, operative vaginal delivery such as vacuum-assisted vaginal delivery or forceps delivery, and excessive abduction of the thighs during delivery [2,3,5]. Clinical presentation may include an audible “pop” at the time of delivery, severe postpartum pelvic pain disproportionate to delivery expectations, labial edema or hematoma, gait disturbance, and inability to ambulate [2,6]. Because this condition is uncommon and symptoms may overlap with more typical postpartum discomfort, diagnosis is frequently delayed.

Management strategies range from conservative treatment with pelvic binders, pain control, and physical therapy to operative stabilization in cases of severe diastasis, pelvic instability, or failure of nonoperative management [4]. Given the rarity of this condition and the lack of standardized management guidelines, multidisciplinary collaboration is essential. This case highlights the diagnostic challenges, decision-making process, and successful multidisciplinary management of severe pubic symphysis rupture following vaginal delivery.

## Case presentation

A multiparous patient underwent an elective induction of labor at term. At delivery, an audible “pop” was heard. Following the delivery, the patient developed severe bilateral labial edema, more pain than expected for a vaginal delivery, and external rotation of both legs. The hemoglobin on postpartum day one decreased by 4 g/dL from the patient’s admission hemoglobin. This decrease could not be explained by the observed blood loss of 250 mL from the otherwise uncomplicated vaginal delivery and was consistent with concealed hemorrhage related to the extrafascial hematoma identified on imaging.

**Figure 1:**
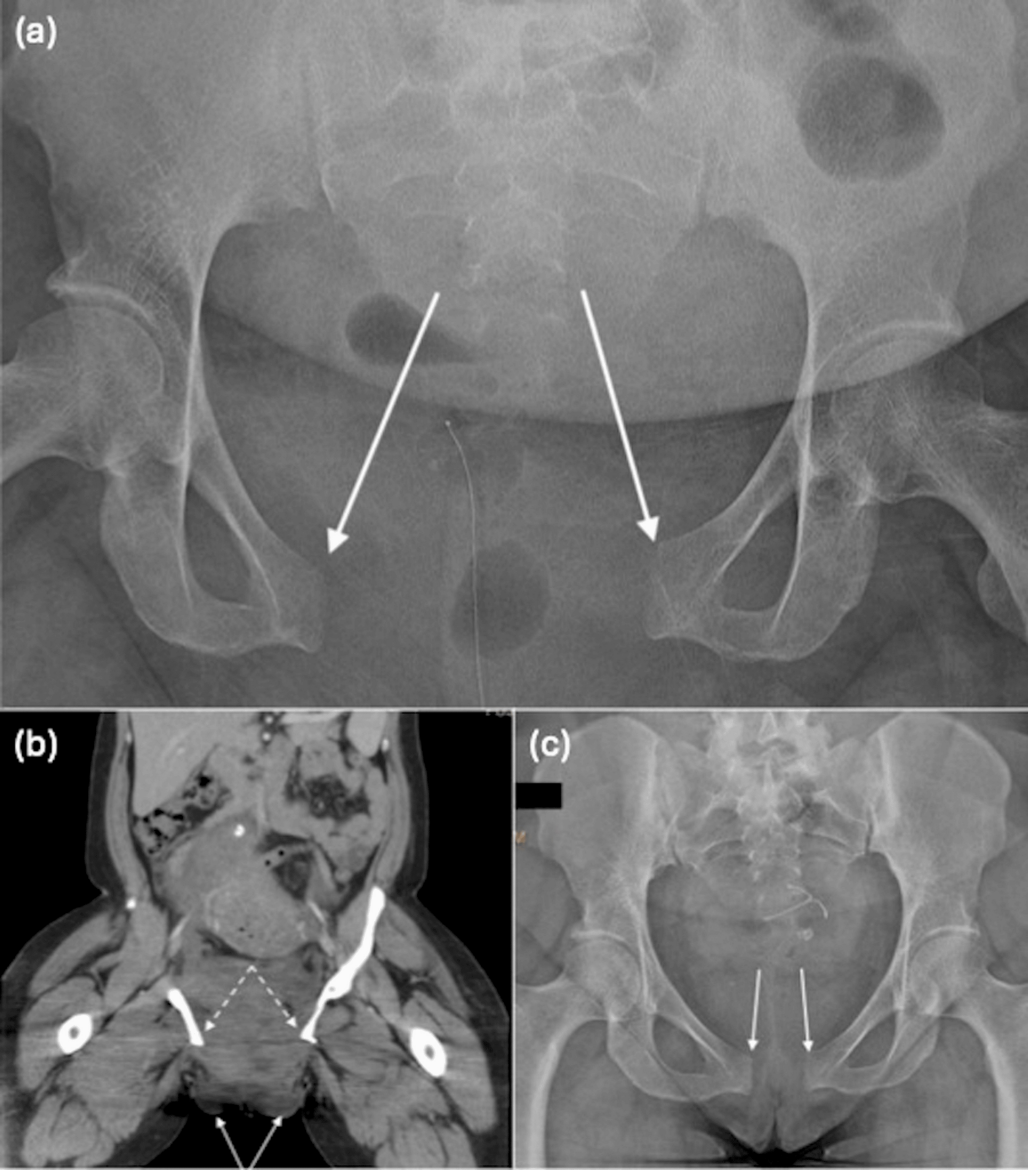
(a) Pelvic radiograph demonstrates widely separated pubic rami (arrows). (b) Computed tomography scan demonstrates labial edema and hematoma (solid arrows) at the level of pubic symphysis rupture with an 8.5 cm pubic symphysis separation (dashed arrows). (c) Pelvic radiograph after surgical management and six months following delivery demonstrates reapproximation of the pubic rami (arrows).

Plain films of the pelvis demonstrated a ruptured pubic symphysis (Figure 1a). Pelvic computed tomography (CT) showed an 8.5 cm separation of the pubic symphysis and an extrafascial hematoma near the pubic symphysis, which extended to both labia (Figure 1b). The orthopedic surgery team initially performed a closed reduction of the pelvic ring by placing an external pelvic binder. The following day, an anterior pelvic ring external fixator was placed after the reduction of the pubic symphysis diastasis. The patient’s hemoglobin remained stable, and her pain was markedly decreased. The patient was discharged with the external fixator as the method of definitive fixation by the orthopedics team as well as outpatient physical and occupational therapy. Weight-bearing and ambulation were limited to walker use. Prior to discharge, informed consent for description of the case was obtained in conjunction with hospital policy, waiving the need for Institutional Review Board (IRB) approval.

The external fixator was removed after approximately seven weeks in an outpatient procedure. Plain film imaging at the time of fixator removal demonstrated symphyseal diastasis of approximately 3 cm. Six months following delivery, the pubic symphysis appeared well-approximated on imaging (Figure 1c); precise measurement at that time was not formally quantified.

## Discussion

Many cases of peripartum pubic symphysis separation may be missed. If there are significant symptoms that do not improve on their own, cases may be managed conservatively with pelvic binders, bed rest, analgesia, and gradual mobilization, and many patients experience symptomatic improvement over weeks to months [2,3,6]. Surgical intervention is generally reserved for patients with severe diastasis, commonly cited as greater than 4 cm, associated pelvic ring instability, refractory pain, or significant impairment in ambulation [4,7]. However, clear thresholds for operative management are not well established, and recommendations are largely based on case reports and small case series.

In cases of severe separation, early orthopedic consultation and stabilization may improve pain control, facilitate mobilization, and reduce long-term morbidity [4,7]. External fixation has been described as an effective method of anterior pelvic ring stabilization, particularly in the acute setting, allowing reduction of the symphyseal gap while minimizing surgical morbidity [8]. In the present case, the degree of separation (8.5 cm), associated hematoma, and functional impairment prompted operative management following initial binder stabilization. Multidisciplinary coordination among obstetrics, radiology, and orthopedic surgery was critical to timely diagnosis and management. Postoperatively, the patient progressed from walker-assisted ambulation to independent mobility, returned to work, and reported no persistent pelvic pain at long-term follow-up.

Long-term outcomes following pubic symphysis rupture are variable. While many patients recover fully, some experience chronic pelvic pain, dyspareunia, or gait abnormalities [3,6]. Early recognition and appropriate management may reduce the risk of persistent symptoms. This case demonstrates that, in selected patients with severe pubic symphysis rupture, operative stabilization can result in favorable functional outcomes, with sustained symptom resolution at long-term follow-up. However, this cannot be accomplished without a high index of suspicion, strong interdisciplinary communication, and a collaborative approach to diagnosis and management. As a single case report, these findings are limited in generalizability, and management decisions must be individualized based on patient presentation and multidisciplinary assessment.

## Conclusions

We recommend a high index of suspicion for pubic symphysis rupture. An unexpected “pop” at delivery and atypical symptoms including isolated labial edema or an unexpected drop in hemoglobin require thorough physical examination and multidisciplinary collaboration between obstetrics, radiology, and orthopedic teams. Physical examination and imaging may lead to an early diagnosis and prompt management of this unusual complication. While plain film imaging remains the appropriate initial study to diagnose pelvic ring injury, further evaluation with a pelvic CT scan is often needed. This case should encourage and serve as an example to demonstrate how excellent communication between services can foster collaboration and ultimately provide the correct diagnosis and timely treatment.
